# Quantitative assessment of hepatic steatosis by ultrasound-guided attenuation parameter in patients with impaired glucose tolerance

**DOI:** 10.1186/s13244-025-02123-1

**Published:** 2025-11-06

**Authors:** Ruixia Gao, Jiahao Han, Danlei Song, Pingping Wang, Huihui Chen, Huiming Shen, Jia Li

**Affiliations:** 1 Medical School, Southeast University, Nanjing, China; 2 Department of Ultrasonography, Zhong Da Hospital, Medical School, Southeast University, Nanjing, China

**Keywords:** Fatty liver, Ultrasound, Magnetic resonance imaging

## Abstract

**Objectives:**

Our objective was to provide further evidence regarding the diagnostic accuracy of ultrasound-guided attenuation parameter (UGAP) for detecting hepatic steatosis in a selected and homogeneous cohort of patients with impaired glucose tolerance using magnetic resonance imaging-proton density fat fraction (MRI-PDFF) as the standard reference method.

**Materials and methods:**

From October 2023 to March 2024, individuals with impaired glucose tolerance and suspected metabolic dysfunction-associated steatotic liver disease (MASLD) who underwent abdominal ultrasound and MRI-PDFF were enrolled in this prospective study. Multivariable linear regression was performed to assess independent factors associated with UGAP. The correlation between UGAP, and MRI-PDFF was evaluated using the Pearson correlation coefficient. The diagnostic performance of the UGAP for hepatic steatosis was assessed using the area under the receiver operating characteristic curve (AUC).

**Results:**

A total of 193 participants (median age, 35 years; IQR, 25–48 years; 98 male) were evaluated, including 90 participants (47%) with MASLD. Body mass index (BMI), waist circumference, and skin-liver capsule distance were independently associated with UGAP. UGAP was strongly and positively correlated with MRI-PDFF (*r* = 0.890, *p* < 0.001). The AUCs of UGAP for diagnosing hepatic steatosis ≥ S1 (MRI-PDFF ≥ 6.4%), ≥ S2 (MRI-PDFF ≥ 17.4%), and ≥ S3 (MRI-PDFF ≥ 22.1%) were 0.980 (95% CI: 0.957–1.000), 0.955 (95% CI: 0.912–0.999), and 0.985 (95% CI: 0.970–1.000), respectively.

**Conclusions:**

UGAP could accurately diagnose hepatic steatosis and estimate the hepatic fat fraction.

**Critical relevance statement:**

UGAP has high diagnostic performance for hepatic steatosis. UGAP offers a method of quantifying liver fat content similar to MRI-PDFF, which is anticipated to be beneficial in the long-term monitoring of patients with impaired glucose tolerance.

**Key Points:**

- Hepatic steatosis is an early factor that causes liver damage in MASLD.
- UGAP has an accurate diagnostic performance for hepatic steatosis.
- UGAP was positively correlated with MRI- proton density fat fraction.
- Some clinical parameters may affect UGAP measurement.

**Graphical Abstract:**

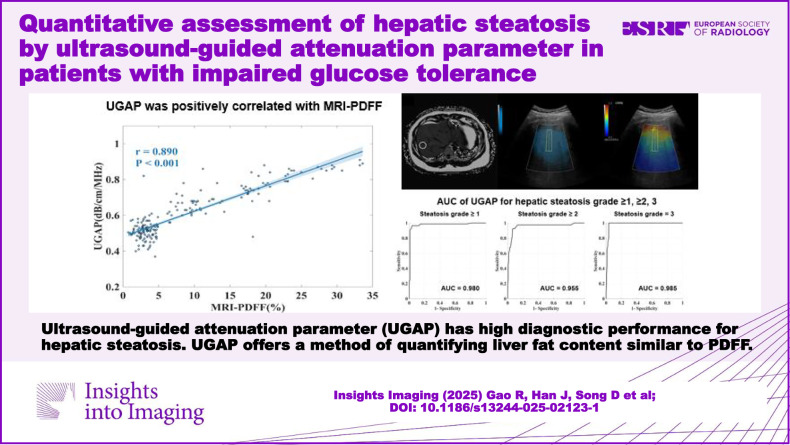

## Introduction

Metabolically-dysfunction-associated steatotic liver disease (MASLD) is currently the most common chronic liver disease worldwide that affects approximately 30% of the global population [1, 2]. MASLD can progress from simple steatosis to necroinflammation, cirrhosis, and even hepatocellular carcinoma [3]. Early diagnosis of MASLD is essential as it may significantly increase the risk of systemic disease and mortality [4]. Hepatic steatosis is the main criterion to define MASLD. Hepatic fat content is acknowledged as a pivotal early factor contributing to liver damage and the advancement of MASLD [5]. Many of the experimental drugs used to treat MASLD intervene with the aim of reducing the amount of hepatic steatosis [6]. In this context, precise quantification of hepatic steatosis plays a central role. Liver biopsy remains the reference standard for diagnosing hepatic fat content [7]. However, liver biopsy is an invasive procedure with the potential for serious complications. Furthermore, liver biopsy is unfeasible for screening and frequent monitoring of liver disease progression [8, 9]. Therefore, there is an urgent need for a non-invasive, reproducible, and cost-effective method for the screening and risk stratification of patients with MASLD.

Magnetic resonance imaging-proton density fat fraction (MRI-PDFF) has been recognized as an alternative to liver biopsy for the reproducible and accurate quantification of hepatic fat content [10–12]. The current MRI-PDFF cut-off value for grading hepatic steatosis is the most commonly used: steatosis grade 0 (S0) < 6.4%; 6.4% ≤ S1 < 17.4%; 17.4% ≤ S2 < 22.1%; S3 ≥ 22.1% [13]. Nevertheless, the implementation of MRI-PDFF is limited by its accessibility and contraindications [10]. Conventional B-mode US is the first-line screening tool for patients at risk for MASLD. The method uses a qualitative grading scale that is low in sensitivity for mild steatosis [8].

In the past few years, US-based techniques have been developed for numerically quantifying hepatic fat content, such as ultrasound attenuation parameter (UAP) and the ultrasound-guided attenuation parameter (UGAP). UAP is a physical parameter obtained with the FibroTouch (Wuxi Hisky Medical Technology Co Ltd) device to quantify hepatic steatosis. FibroTouch uses a dynamic broadband ultrasound probe to assess liver conditions in normal-weight, overweight, and obese individuals [14]. Among the US-based techniques for the assessment of hepatic steatosis, the UGAP has been demonstrated to be closely correlated with MRI-PDFF [15, 16], emerging as a reproducible and easy-to-perform ultrasound imaging technique that quantifies hepatic steatosis [17].

In this work, our objective was to provide further evidence regarding the diagnostic accuracy of UGAP for detecting hepatic steatosis in a selected and homogeneous cohort of patients with impaired glucose tolerance using MRI-PDFF as the standard reference method.

## Materials and methods

### Study participants

This prospective study was conducted in a single center in China on 206 consecutive patients with impaired glucose tolerance who underwent abdominal ultrasound and MRI-PDFF between October 2023 and March 2024. Inclusion criteria were as follows: age 18–85-years-old and having impaired glucose tolerance. Exclusion criteria were as follows: (1) pregnant; (2) incomplete clinical information; (3) contraindications for MRI-PDFF; (4) unable to adhere to a 6-h fast prior to the MRI and ultrasound tests; (5) severe comorbidities such as cardiovascular, hepatic, renal, immunological, psychiatric disorders, or other serious conditions; (6) due to the presence of large cysts, hemangiomas and other liver lesions, the area of the liver that can be detected by ultrasound is smaller than that required for UGAP measurement; and (7) chronic viral hepatitis, human immunodeficiency virus infection, excessive alcohol consumption (i.e., pre-screening weekly intake 140–350 g women, 210–420 g men) or other causes of chronic liver disease (e.g., Wilson’s disease, hemochromatosis, autoimmune liver diseases) or drug-induced liver injury (e.g., amiodarone, methotrexate, and systemic corticosteroids).

Over the course of three days, the participants underwent a series of standardized clinical evaluations, including a medical history, anthropometric examination, and biochemical testing. Additionally, they underwent UGAP, UAP, and MRI-PDFF.

In accordance with the clinical data and MRI-PDFF, the MRI-PDFF values less than 6.4% were included in the non-MASLD group [13]. Conversely, the MRI-PDFF values greater than or equal to 6.4% were included in the MASLD group [2, 13].

The study involving human participants was performed in accordance with the Declaration of Helsinki and approved by the IEC for Clinical Research of Zhongda Hospital, Affiliated to Southeast University (no. 2023ZDSYLL116-P01). All study participants provided informed written consent. The study protocol was registered in the Chinese Clinical Trial Registry, and the registration number was ChiCTR2300076457.

### UGAP measurements

The UGAP measurement was performed using a LOGIQ E20 ultrasound system (General Electric Healthcare) with a C1-6-D probe. Previous studies have demonstrated that UGAP shows excellent intra- and inter-observer reproducibility [15, 16, 18–20]. Therefore, the ultrasound examinations were performed by one radiologist with over four years of experience in abdominal ultrasound, who was blinded to the participant’s UAP, MRI-PDFF, and clinical data. All ultrasound (US) measurements were performed after participants fasted for no less than 6 h in a supine or light left lateral decubitus (< 30°) position with the right arm in maximal abduction, using the intercostal approach at the right hepatic lobe. Moving the sampling frame (4–8 cm from the hepatic peritoneum) on the large, color-coded attenuation map in the right liver lobe (segment Ⅷ) was automatically adjusted by the system, avoiding intrahepatic vascular, biliary, and other ductal structures. Moreover, selecting the best image to obtain attenuation coefficient (AC) measurements under patient breath-hold conditions. Failure measurements were defined as a red-marked region of interest (ROI) in the display attenuation map. In accordance with the manufacturer’s instructions, at least 12 measurements were performed. The quality criteria included a ratio of the interquartile range (IQR) of the attenuation value to the median being < 30%. The mean of the 12 measurements of AC for each patient was used for statistical analysis. UGAP values were expressed in dB/cm/MHz. All measurements of UGAP, UAP, and MRI-PDFF were performed on patients within three days.

### UAP measurements

UAP measurement was obtained using the FibroTouch-Pro2500 (Wuxi Hisky Medical Technology Co. Ltd.) by a well-trained nurse (performed more than 500 examinations). The nurse was blinded to the participant’s UGAP, MRI-PDFF, and clinical data. All individuals were fasted for at least 6 h before the examination. The measurements were performed on the right lobe of the liver through the seventh to ninth intercostal spaces. The UAP measurement was expressed in dB/m. Only examinations with at least ten successful acquisitions, with an IQR/median of UAP ≤ 30%, and with a success rate ≥ 60% were deemed valid.

### Magnetic resonance imaging-based proton density fat fraction measurements

MRI was performed using a 3-T system (Siemens Healthineers). Hepatic steatosis was evaluated using MRI-PDFF by a multiecho-Dixom method (vibe_q-dixon sequence) with the following parameters: scanning area included the whole liver, TR/TE = 9.00/1.05/2.46/3.69/4.92/6.15/7.38 ms; echo time = six echoes 1.3 ms; field of view (280 × 320 mm); capture matrix (111 × 160); reconstruction matrix (640 × 640); flip angle 4 angle; slice thickness (3.5 mm); capture voxel (2.8 × 2.8 × 3.5 mm); reconstruction voxel (1.4 × 1.4 × 3.5 mm); acquisition time = 16 s. PDFF was measured using the average of the six regions of interest (ROI) (20 × 20 × 20 mm^3^, deviation < 10 mm^3^) placed in liver segment VIII, avoiding the bile ducts and blood vessels, by one hepatology radiologist with over ten years’ experience, blinded to the participant’s UGAP, UAP, and clinical data.

The MRI-PDFF cut-off values for diagnosing steatosis grades, as reported by An Tang et al, are as follows: steatosis: grade 0 was defined as MRI-PDFF < 6.4%; grade 1 as 6.4% ≤ MRI-PDFF < 17.4%; grade 2 as 17.4% ≤ MRI-PDFF < 22.1%; grade 3 as MRI-PDFF ≥ 22.1% [13]. Considering that different cutoff values for MRI-PDFF to grading hepatic steatosis were concluded in different research, the diagnostic performance of UGAP was also confirmed using different diagnostic criteria (5.2%, 11.3%, and 17.1% [21]; 5.5%, 15.5%, and 20.5% [22]).

### Statistical analysis

The sample size was calculated using the confidence intervals (CI) for the Area Under an ROC Curve model of PASS15. PASS15 is a statistical software for efficacy analysis and sample size estimation. Considering that this study was a diagnostic trial and did not necessitate relevant interventions or follow-up of patients, a loss to follow-up rate of 5% was set. A CI width of 0.10, α = 0.05, an expected area under the receiver operating characteristic curve (AUC) of 0.90, and enrolled MASLD patients: patients without hepatic steatosis = 1:1. 84 subjects needed to be enrolled in each of the two final groups [23, 24].

Statistical analyses were performed using SPSS (version 27.0) and MATLAB 2023b (Mathworks, Inc.) software. Statistical significance was defined as *p* < 0.05.

Continuous variables were expressed as mean (standard deviation [SD]) or median (IQR), and categorical variables as percentages. Pearson’s correlation analysis or Spearman’s correlation analysis was used to compare UGAP with various parameters and UAP with MRI-PDFF. Independent influences on UGAP using multiple linear regression analysis. Kruskal–Wallis test was used to compare UGAP measurement distribution according to steatosis grades. A receiver operating characteristic analysis was performed to assess the diagnostic performance of UGAP and UAP for the diagnosis of steatosis. The optimal cut-off value for UGAP and UAP was determined using the Youden index to obtain the sensitivity, specificity, positive predictive value (PPV), and negative predictive value (NPV). The AUC of UGAP and UAP for the diagnosis of MRI-PDFF steatosis was compared using a DeLong test.

## Results

### Participant characteristics

**Fig. 1:**
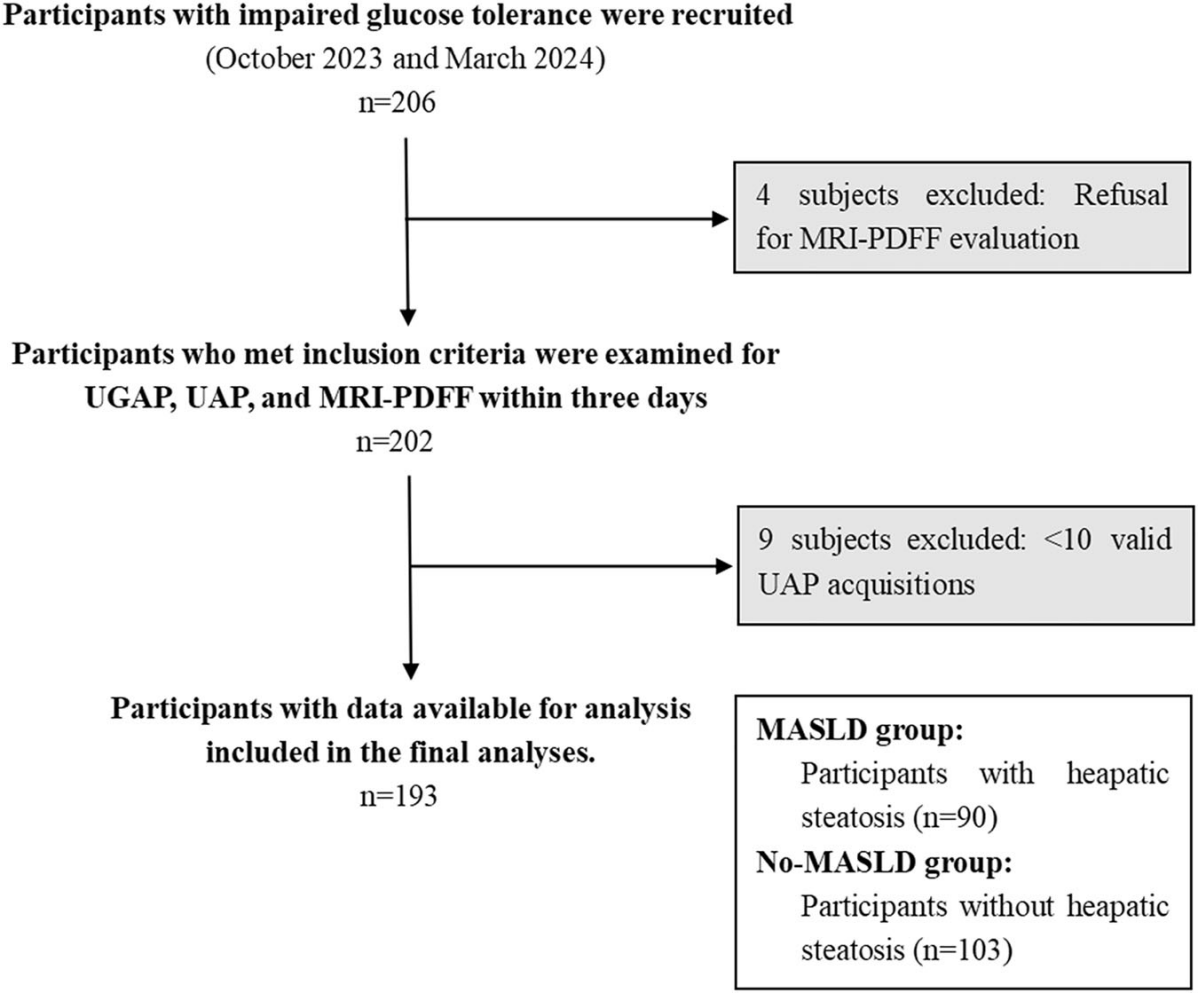
Inclusion and exclusion flowchart of eligible participants. MASLD, metabolic-associated fatty liver disease; UGAP, ultrasound-guided attenuation parameter; MRI-PDFF, magnetic resonance imaging-proton density fat fraction

**Fig. 2:**
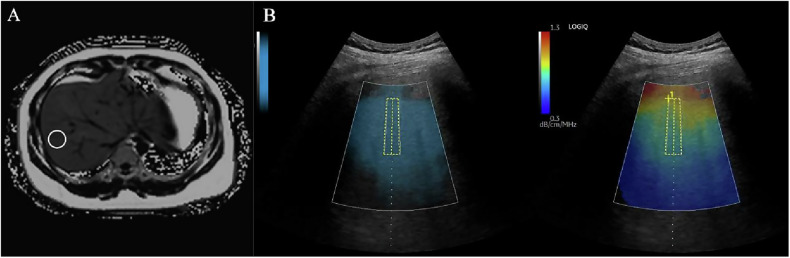
UGAP and MRI in a 48-year-old male patient with MASLD and whose BMI was 29.7. **A** The ROI of the MRI-PDFF was illustrated. A single ROI (20 × 20 × 20 mm^3^) was placed for PDFF measurement in liver segment VIII. The displayed PDFF value is 28.4%. **B** The quality image (left) shows the liver parenchyma to select the best image for the AC measure. The attenuation image (UGAP) (right) provides a quantitative and visual depiction of the attenuation of the right lobe of the liver, utilizing real-time color-coded maps. The degree of attenuation within the specified sampling frame is indicated by a color code. Red indicates a high AC, and blue indicates a low AC. The AC value displayed in the figure is 0.86 dB/cm/MHz

From October 2023 to March 2024, 206 consecutive participants were enrolled in the study. Of those participants, 13 individuals were excluded because of refusal of MRI-PDFF evaluation (*n* = 4) and < 10 valid UAP acquisitions (*n* = 9). One hundred ninety-three participants with valid UGAP, UAP, and PDFF measurements were finally evaluated (median age, 35 years; IQR, 25–48 years; 98 male). The participants were classified into the MASLD group (*n* = 90) and non-MASLD group (*n* = 103) according to the cutoff PDFF value of 6.4% (Figs. 1 and 2). Participants’ baseline characteristics are displayed in Table 1. The median (IQR) values of UGAP, UAP, and MRI-PDFF in the overall cohort were 0.56 (0.51–0.71) dB/cm/MHz, 256.0 (219.0–293.0) dB/m, and 4.50 (2.92–13.40)%, respectively. The median (IQR) values of body mass index (BMI), waist circumference, skin-liver capsule distance, AST, ALT, GGT, Triglycerides, hs-CRP, UGAP, UAP and MRI-PDFF in the MASLD group were 27.0 (24.4–31.0) kg/m^2^, 92.0 (87.0–101.5) cm, 19.2 (17.5–20.7) mm, 22.0 (17.0–29.5) U/L, 24.0 (18.0–37.0) U/L, 31.0 (24.0–45.5) U/L, 2.00 (1.45–3.81) mmol/L, 1.21 (0.81–4.09) mg/L, 0.72 (0.67–0.81) dB/cm/MHz, 296.0 (274.0–316.5) dB/m and 14.90 (9.75–20.60)%, which was higher than in the non-MASLD group. The median (IQR) values of HDL-C in the MASLD group were 1.18 (0.89–1.54) mmol/L, lower than in the non-MASLD group. The data presented above show the characteristics of the MASLD.

**Table 1:** Clinical characteristics and laboratory data of the participants

Characteristic	Total patients (*n* = 193)	Non-MASLD (*n* = 103)	MASLD (*n* = 90)	*p* value
Age, years	35.0 (25.0–48.0)	27.0 (24.0–42.0)	45.0 (34.0–54.0)	**< 0.001**
Male/female	98/95	38/65	60/30	**<** **0.001**
BMI, kg/m^2^	23.5 (21.2–27.1)	21.6 (20.0–23.5)	27.0 (24.4–31.0)	**<** **0.001**
Waist circumference, cm	82.0 (72.0–91.5)	73.5 (68.0–81.0)	92.0 (87.0–101.5)	**< 0.001**
Skin-liver capsule distance, mm	16.8 (14.5–19.1)	14.8 (13.5–16.7)	19.2 (17.5–20.7)	< **0.001**
Platelets (×10^9^/L)	236.0 (203.5–263.0)	236.0 (209.0–253.3)	236.0 (198.0–278.0)	0.733
AST, U/L	17.0 (14.0–22.0)	15.0 (13.0–18.0)	22.0 (17.0–29.5)	**< 0.001**
ALT, U/L	18.0 (14.5–24.0)	16.0 (12.0–18.0)	24.0 (18.0–37.0)	**<** **0.001**
GGT, U/L	29.0 (19.0–39.0)	27.5 (18.0–35.0)	31.0 (24.0–45.5)	**<** **0.001**
Albumin, g/L	45.6 (39.8–47.9)	45.7 (38.7–47.7)	45.2 (41.2–48.2)	0.481
Total Bilirubin, μmol/L	10.5 (8.2–13.5)	11.0 (8.5–14.4)	10.1 (7.9–13.1)	0.150
Triglycerides, mmol/L	1.21 (0.82–1.88)	0.98 (0.75–1.21)	2.00 (1.45–3.81)	**<0.001**
Total cholesterol, mmol/L	5.05 (4.49–5.65)	4.98 (4.61–5.51)	5.12 (4.12–5.90)	0.998
HDL-C, mmol/L	1.34 (1.11–1.52)	1.37 (1.24–1.52)	1.18 (0.89–1.54)	**<0.001**
LDL- C, mmol/L	2.68 (2.32–2.97)	2.68 (2.47–2.92)	2.68 (2.11–3.05)	0.861
hs-CRP, mg/L	0.81 (0.78–1.58)	0.81 (0.78–1.01)	1.21 (0.81–4.09)	**<0.001**
UGAP, dB/cm/MHz	0.56 (0.51–0.71)	0.52 (0.48–0.54)	0.72 (0.67–0.81)	**<** **0.001**
UAP, dB/m	256.0 (219.0–293.0)	224.5 (202.2–255.0)	296.0 (274.0–316.5)	**<** **0.001**
MRI-PDFF, %	4.50 (2.92–13.40)	3.1 (2.43–3.87)	14.90 (9.75–20.60)	**<** **0.001**
Steatosis grade according to MRI-PDFF, S1/S2/S3/, *n*^a^	82.0 (72.0–91.5)	73.5 (68.0–81.0)	46/21/23	**< 0.001**

Values are mean (standard deviation), median (IQR), or *n* (%)

*p* value determined by comparing characteristics of patients without MASLD (MRI-PDFF < 6.4%) and with MASLD (MRI-PDFF ≥ 6.4%)

Bold indicates significant *p* values < 0.05

Skin-liver capsule distance for the body in the supine position

*AST* aspartate aminotransferase, *ALT* alanine aminotransferase, *GGT* γ-glutamyltransferase, *HDL-C* high-density lipoprotein cholesterol, *LDL-C* low-density lipoprotein cholesterol, *hs-CRP* hypersensitive C-reactive protein, *UGAP* ultrasound-guided attenuation parameter, *UAP* ultrasound attenuation parameter, *MRI-PDFF* magnetic resonance imaging-proton density fat fraction

^a^MRI-PDFF thresholds for the grades of steatosis were 6.4%, 17.4%, and 22.1% for S1, S2, and S3, respectively

### Association of UGAP with various parameters

Correlations between various parameters and UGAP values are shown in Table 2. Univariate analysis revealed significant correlations between UGAP and other factors, including age, sex, BMI, waist circumference, skin-liver capsule distance, AST, ALT, GGT, triglycerides, and hs-CRP. Multivariate linear regression analysis showed that the independent influences of UGAP were BMI, waist circumference, and skin-liver capsule distance.

**Table 2:** Correlation between UGAP and clinical characteristics (*n* = 193)

Variable	Correlation coefficient (*r*)	Univariate analysis (*p* value)	Multivariate analysis (*p* value)
Age, years	0.381	**< 0.001**	0.170
Male/female	−0.227	**<** **0.001**	0.293
BMI, kg/m^2^	0.635	**<** **0.001**	**< 0.001**
Waist circumference, cm	0.675	**< 0.001**	**0.003**
Skin-liver capsule distance, mm	0.660	**<** **0.001**	**<** **0.001**
Platelets (10^9^/L)	0.046	0.526	0.185
AST, U/L	0.592	**< 0.001**	0.529
ALT, U/L	0.508	**<** **0.001**	0.269
GGT, U/L	0.282	**<** **0.001**	0.351
Albumin, g/L	0.091	0.208	0.485
Total Bilirubin, μmol/L	−0.120	0.095	0.796
Triglycerides, mmol/L	0.638	**< 0.001**	0.122
Total cholesterol, mmol/L	−0.034	0.642	0.067
HDL-C, mmol/L	−0.137	0.058	0.053
LDL-C, mmol/L	−0.038	0.604	0.113
hs-CRP, mg/L	0.384	**< 0.001**	0.123

Bold indicates significant *p* values < 0.05

*AST* aspartate aminotransferase, * ALT* alanine aminotransferase, *GGT* γ-glutamyltransferase, *HDL-C* high-density lipoprotein cholesterol, *LDL-C* low-density lipoprotein cholesterol, *hs-CRP* hypersensitive C-reactive protein

### Correlation between UGAP, UAP, and MRI-PDFF

**Fig. 3:**
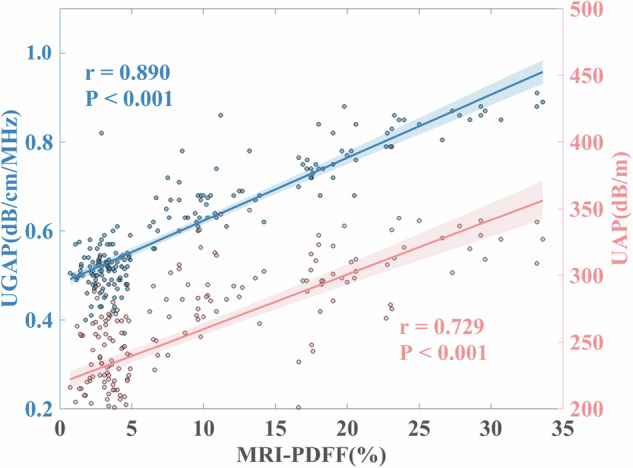
Scatterplots show the correlation between UGAP, UAP, and MRI-PDFF. UGAP, ultrasound-guided attenuation parameter; UAP, ultrasound attenuation parameter; MRI-PDFF, magnetic resonance imaging-proton density fat fraction

A very high positive correlation was found between the measured AC with UGAP and MRI-PDFF (%) (*r* = 0.890, *p* < 0.001). A high positive correlation was found between UAP and MRI-PDFF (%) (*r* = 0.729, *p* < 0.001) (Fig. 3).

### Diagnostic performance of UGAP and UAP

**Fig. 4:**
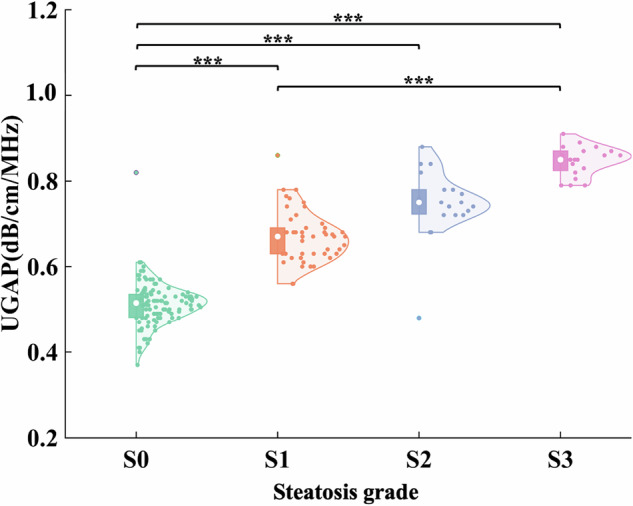
UGAP values distribution for steatosis grades classified by MRI-PDFF. UGAP, ultrasound-guided attenuation parameter; MRI-PDFF, magnetic resonance imaging-proton density fat fraction. ***Significant difference (*p* < 0.001)

The participants were classified into four categories (hepatic steatosis grades 0–3) based on the reference method of MRI-PDFF. The median UGAP values of patients with S0, S1, S2, and S3 were 0.52, 0.67, 0.75, and 0.85 dB/cm/MHz, respectively. Pairwise comparison showed a significant difference between S0 and all other steatosis grades and between S1 and S3 (*p* < 0.001) (Fig. 4).

**Fig. 5:**
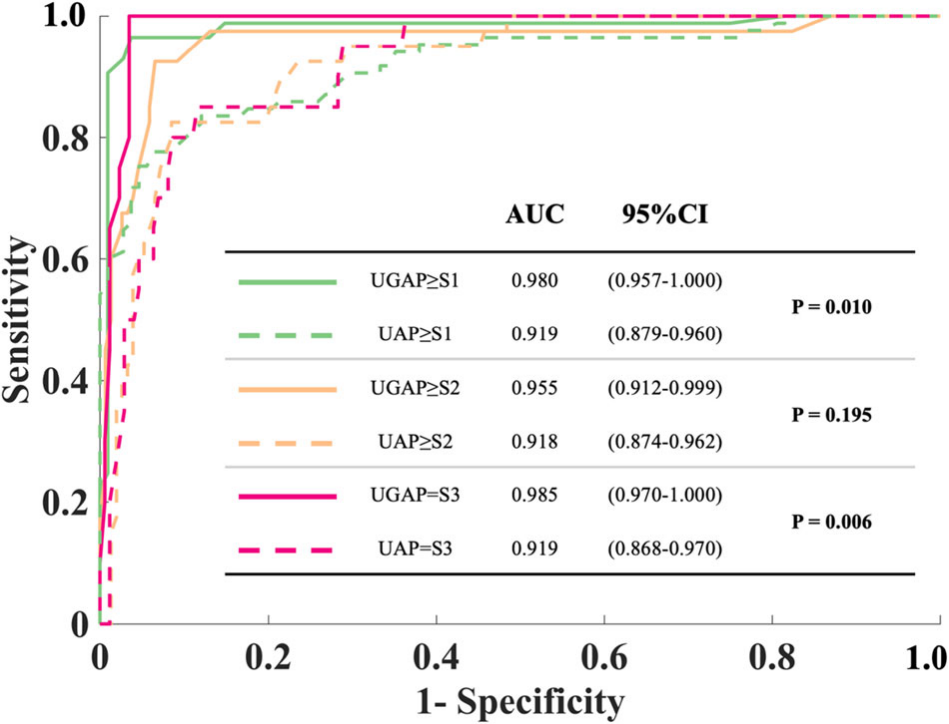
Receiver operating characteristic curve for UGAP and UAP in the diagnosis of hepatic steatosis. AUC, area under the receiver-operating characteristic curve; CI, confidence interval; UGAP, ultrasound-guided attenuation parameter; UAP, ultrasound attenuation parameter

The diagnostic performance of UGAP and UAP for MRI-PDFF assessed hepatic steatosis are presented in Table 3. The comparison of UGAP and UAP AUCs are provided in Fig. 5. The UGAP AUCs for steatosis grade ≥ 1, ≥ 2, and = 3 were 0.980 (95% CI: 0.957–1.000), 0.955 (95% CI: 0.912–0.999), and 0.985 (95% CI: 0.970–1.000), respectively. The optimal cut-off values for UGAP for steatosis grade ≥ 1, ≥ 2, and = 3 were 0.60, 0.72, and 0.79 dB/cm/MHz, respectively (Table 3). UGAP outperformed UAP for the diagnosis of steatosis (≥ S1 [*p* = 0.010] and S3 [*p* = 0.006]). The result was supported by the assessment of other PDFF thresholds (Table S1). Moreover, the impact of BMI, waist circumference, and skin-to-liver surface distance on the diagnostic efficacy of UGAP steatosis was assessed. The Table S2 displays the AUCs for different subgroups of steatosis grades ≥ 1, ≥ 2, and = 3 [25].

**Table 3:** Diagnostic ability and optimal cutoff values of UGAP and UAP for grading hepatic steatosis using MRI-PDFF as reference

Steatosis grade	AUC (95% CI)	Cutoff value	Sensitivity (%)	Specificity (%)	PPV (%)	NPV (%)
UGAP (dB/cm/MHz)						
≥ S1	0.980 (0.957–1.000)	0.60	96.3	96.5	95.34	97.19
≥ S2	0.955 (0.912–0.999)	0.72	93.5	92.5	76.74	95.33
S3	0.985 (0.970–1.000)	0.79	100.0	96.5	75.00	98.82
UAP (dB/m)						
≥ S1	0.919 (0.879–0.960)	266.5	88.0	83.5	84.52	87.14
≥ S2	0.918 (0.874–0.962)	294.0	82.5	91.5	69.53	94.50
S3	0.919 (0.868–0.970)	301.5	85.0	88.4	45.95	98.12

Values are *n* (%), unless otherwise indicated

*AUC* area under the receiver-operating characteristic curve, *CI* confidence interval, *UGAP* ultrasound-guided attenuation parameter, *UAP* ultrasound attenuation parameter, *MRI-PDFF* magnetic resonance imaging-proton density fat fraction, *PPV* positive predictive value, *NPV* negative predictive value

## Discussion

In this prospective study, UGAP demonstrated high diagnostic performance for hepatic steatosis in patients with impaired glucose tolerance and MASLD, using MRI-PDFF as the standard reference. UGAP was strongly positively correlated with MRI-PDFF. Furthermore, BMI, waist circumference, and skin-liver capsule distance were independently associated with UGAP. Finally, this study demonstrates that UGAP was better than UAP for the diagnosis of steatosis, whereas the two methods performed similarly for the diagnosis of moderate steatosis.

The diagnostic performance of UGAP for hepatic steatosis was excellent when MRI-PDFF was used as a reference standard. Some studies have shown that UGAP also has similar diagnostic performance when using histology or controlled attenuation parameter (CAP) as a reference standard [15, 18–20]. However, liver biopsy is limited by its invasiveness, sampling bias, and complications (e.g., bleeding) and is unfeasible for screening and frequent monitoring of liver disease progression [8, 9]. At present, MRI-PDFF is considered to be the more accurate and robust non-invasive method. The PDFF measurement is straightforward to perform by identifying multiple regions of interest on the liver parenchyma [10–12]. The regions of interest for both PDFF and UGAP in our study were obtained from the S8 segment of the liver. MRI-PDFF was previously reported to be superior to CAP for diagnosing steatosis grades in non-alcoholic fatty liver disease (NAFLD) patients using liver biopsy as the reference [21]. Accordingly, MRI-PDFF appears to be a precise, quantitative, and reproducible method for the diagnosis of steatosis.

Following the revision of the exclusive diagnosis of NAFLD to a positive diagnosis of MASLD, there was a notable change in the population characteristics [2]. In previous studies, the population may comprise heterogeneous cohorts with NAFLD and other causes of chronic liver diseases [15, 16, 18–20]. Therefore, different etiologies may have an impact on the UGAP measurement of hepatic steatosis. In our study, for the MASLD population, the overall diagnostic accuracy of UGAP in distinguishing any steatosis grade was excellent with an AUC of 0.955–0.985. The cutoff values for predicting steatosis grades 1, 2, and 3 were 0.60, 0.72, and 0.79 dB/cm/MHz, respectively. In a recent study of a large cohort with chronic liver disease, using MRI-PDFF as the reference, UGAP showed AUCs of 0.910, 0.912, and 0.894 for diagnosing steatosis grades 1, 2, and 3, respectively. In the subgroup analysis, the diagnostic accuracy of UGAP in the NAFLD group showed an AUC of 0.834–0.898 [15]. In contrast, the results obtained in our study are more relevant and applicable to patients with MASLD. Moreover, our study’s sample size was meticulously calculated to guarantee the diagnostic precision of UGAP.

Furthermore, it should be considered that the MRI-PDFF cut-off values for diagnosing steatosis in the literature may exhibit slight discrepancies (e.g., 6.4%, 17.4% and 22.1% [13], 5.2%, 11.3% and 17.1% [21]; 5.5%, 15.5% and 20.5% [22]). Therefore, our study evaluated the impact of several alternative thresholds. The AUCs of the alternative MRI-PDFF cut-off values for different grades of hepatic steatosis were greater than 0.950. It was demonstrated that UGAP has a similar diagnostic value at different thresholds.

Recently, several studies showed that BMI was independently associated with UGAP values [15, 26]. Severe obesity may affect the ability of UGAP to diagnose grade 3 steatosis. The AUC for UGAP diagnosis of grade 3 steatosis in the subgroup with a BMI ≥ 30 kg/m^2^ was 0.776, significantly lower than the other grades [15]. Similarly, our study showed that BMI (*p* < 0.001), waist circumference (*p* = 0.003), and skin-liver capsule distance (*p* < 0.001) were independently associated, as illustrated in Table 2. Individuals with elevated BMI and waist circumference typically exhibit a more substantial subcutaneous adipose tissue [25]. The penetration of ultrasound sound waves into thicker subcutaneous adipose tissues may result in increased distortion of the radiofrequency signal, which could have a detrimental impact on the estimation of quantitative ultrasound attenuation [17]. Subgroup analyses of BMI, waist circumference, and skin-liver capsule distance were performed in our study, and the diagnostic accuracy of apparent UGAP for different subgroups of steatosis severity may not be influenced by independent factors, as the AUC was higher than 0.800.

In addition to these findings, we conducted a comparative analysis of UGAP and UAP, which showed that UGAP outperformed UAP in the diagnosis of hepatic steatosis. This can be interpreted by UGAP conventional real-time B-mode imaging of the liver target area, providing high-quality images for measurement and avoiding interference from ductal structures or artifacts. In contrast, UAP can only measure in A-mode.

In our study, among the AUCs of different liver steatosis grades in UGAP, the AUC of S2 was relatively low. This phenomenon may be attributed to the limited proportion of the S2 sample size in the MASLD group, as evidenced by the literature on the distribution of hepatic steatosis grades in the Chinese population [27]. However, it should be noted that the sample size of the MASLD group was accurately calculated, and the diagnostic accuracy of UGAP is reliable.

UGAP offers a method of quantifying liver fat content similar to PDFF, which is anticipated to be beneficial in the long-term monitoring of patients with impaired glucose tolerance, enabling the observation of alterations in their liver fat content over time or the assessment of therapeutic interventions. Nevertheless, this hypothesis requires confirmation through prospective long-term studies.

Our study had several limitations. First, this study is a single-center trial with a relatively small sample size. However, as a prospective trial with consecutive enrolment, the sample size was rigorously calculated. It can therefore be concluded that UGAP is an effective diagnostic method for MASLD. Second, the UGAP used in this study is proprietary to the vendor and can only be used with GE scanners. Third, given that only individuals of Chinese descent were included from a single center, the resulting findings may not be applicable to other populations in general.

In summary, the findings of our study indicate that UGAP may represent a simple and accurate approach for quantifying hepatic steatosis in clinical practice.

## ELECTRONIC SUPPLEMENTARY MATERIAL

ELECTRONIC SUPPLEMENTARY MATERIAL

## Abbreviations

AUC: Area under the receiver operating characteristic curve
BMI: Body mass index
CAP: Controlled attenuation parameter
CI: Confidence interval
IQR: Interquartile range
MASLD: Metabolic dysfunction-associated steatotic liver disease
MRI-PDFF: Magnetic resonance imaging-proton density fat fraction
NAFLD: Non-alcoholic fatty liver disease
UAP: Ultrasound attenuation parameter
UGAP: Ultrasound-guided attenuation parameter

## Declarations

### Methodology

- Prospective
- Diagnostic or prognostic study
- Performed at one institution
