# Supplementary material for: Quantitative assessment of hepatic steatosis by ultrasound-guided attenuation parameter in patients with impaired glucose tolerance
Source: Insights Imaging. 2025 Nov 6;16:247. doi: 10.1186/s13244-025-02123-1 (PMC12592611; doi:10.1186/s13244-025-02123-1)
Supplement: Supplementary file 1 — ELECTRONIC SUPPLEMENTARY MATERIAL [file 13244_2025_2123_MOESM1_ESM.pdf]

Quantitative assessment of hepatic steatosis by ultrasound-guided attenuation parameter in patients with impaired glucose tolerance

ELECTRONIC SUPPLEMENTARY MATERIAL

Table S1: Diagnostic Performance of UGAP and UAP for the Diagnosis of Alternative MRI-PDFF thresholds-assessed Hepatic Steatosis

Alternative MRI-PDFF thresholds	UGAP AUC (95%CI)	UAP AUC (95%CI)	P-value
Diagnosis of >5.2% MRI-PDFF(n=91)	0.980(0.958-1.000)	0.921(0.881-0.961)	0.011
Diagnosis of >11.3% MRI-PDFF(n=57)	0.958(0.992-0.993)	0.905(0.856-0.953)	0.063
Diagnosis of >17.1% MRI-PDFF(n-45)	0.958(0.917-1.000)	0.918(0.876-0.951)	0.152
Diagnosis of >5.5% MRI-PDFF(n=91)	0.980(0.958-1.000)	0.921(0.881-0.961)	0.011
Diagnosis of >15.5% MRI-PDFF(n=45)	0.963(0.924-1.000)	0.892(0.837-0.948)	0.031
Diagnosis of >20.5% MRI-PDFF(n=24)	0.977(0.955-0.998)	0.920(0.874-0.966)	0.009

AUC, area under the receiver-operating characteristic curve; CI, confidence interval; UGAP, ultrasound-guided attenuation parameter; UAP, ultrasound attenuation parameter; MRI-PDFF, magnetic resonance imaging-proton density fat fraction.

Table S2: Diagnostic Ability for Grading Hepatic Steatosis Divided by Category Associated With UGAP

Category	Subgroup	AUC		
		Steatosis Grade ≥1	Steatosis Grade ≥2	Steatosis Grade =3
BMI	< 24.0kg/m2	0.980	0.994	0.995
	24.0 - 28.0kg/m2	0.986	0.943	0.979
	≥ 28.0kg/m2	0.986	0.861	0.958
Waist circumference	Male <85cm	0.985	0.997	1.000
	≥85cm	0.921	0.886	0.963
	Female <80cm	0.998	0.987	0.965
	≥80cm	0.989	0.945	0.964
Skin-to-liver Surface distance	< 14.5mm	0.990	0.991	0.995
	14.5-19.1mm	0.959	0.908	0.987
	≥ 19.1mm	0.992	0.926	0.955

AUC, area under the receiver-operating characteristic curve; BMI, body mass index.
